# Safety assessment of five candidate probiotic lactobacilli using comparative genome analysis

**DOI:** 10.1099/acmi.0.000715.v4

**Published:** 2024-01-31

**Authors:** Patrick Josemaria d.R Altavas, Mia Beatriz C. Amoranto, Sang Hoon Kim, Dae-Kyung Kang, Marilen P. Balolong, Leslie Michelle M. Dalmacio

**Affiliations:** ^1^​ Department of Biochemistry and Molecular Biology, College of Medicine, University of the Philippines Manila, Ermita, Manila 1000, Philippines; ^2^​ Department of Animal Resources Science, College of Biotechnology and Bioengineering, Dankook University, Gyeonggi-do, Republic of Korea; ^3^​ Department of Biology, College of Arts and Sciences, University of the Philippines Manila, Ermita, Manila 1000, Philippines

**Keywords:** bioinformatics, comparative genomics, *Lactobacillus*, pathogenicity, probiotics, virulence

## Abstract

Micro-organisms belonging to the *Lactobacillus* genus complex are often used for oral consumption and are generally considered safe but can exhibit pathogenicity in rare and specific cases. Therefore, screening and understanding genetic factors that may contribute to pathogenicity can yield valuable insights regarding probiotic safety. *Limosilactobacillus mucosae* LM1, *Lactiplantibacillus plantarum* SK151, *Lactiplantibacillus plantarum* BS25, *Limosilactobacillus fermentum* SK152 and *Lactobacillus johnsonii* PF01 are current probiotics of interest; however, their safety profiles have not been explored. The genome sequences of LM1, SK151, SK152 and PF01 were downloaded from the NCBI GenBank, while that of *L. plantarum* BS25 was newly sequenced. These genomes were then annotated using the Rapid Annotation using Subsystem Technology tool kit pipeline. Subsequently, a command line blast was performed against the Virulence Factor Database (VFDB) and the Comprehensive Antibiotic Resistance Database (CARD) to identify potential virulence factors and antibiotic resistance (AR) genes. Furthermore, ResFinder was used to detect acquired AR genes. The query against the VFDB identified genes that have a role in bacterial survivability, platelet aggregation, surface adhesion, biofilm formation and immunoregulation; and no acquired AR genes were detected using CARD and ResFinder. The study shows that the query strains exhibit genes identical to those present in pathogenic bacteria with the genes matched primarily having roles related to survival and surface adherence. Our results contribute to the overall strategies that can be employed in pre-clinical safety assessments of potential probiotics. Gene mining using whole-genome data, coupled with experimental validation, can be implemented in future probiotic safety assessment strategies.

## Data Summary

The whole-genome sequence data of *Limosilactobacillus mucosae* LM1, *Lactiplantibacillus plantarum SK151*, *Limosilactobacillus fermentum* SK152 and *Lactobacillus johnsonii* PF01 were obtained using the National Center for Biotechnology Information GenBank with the following accession numbers: CP011013.1, CP030105.1, CP016803.1 and CP024781.1, respectively. *Lactiplantibacillus plantarum* BS25, previously isolated from *balao-balao* (Philippine fermented rice-fish mixture), was sequenced by the Philippine Genome Center, and the genome was deposited at DDBJ/ENA/GenBank under the GenBank accession number: JAVBIR000000000.

The authors confirm all supporting data, code and protocols have been provided within the article or through supplementary data files.

**Impact Statement**

Probiotics are micro-organisms that are often incorporated in fermented foods and supplements due to their intended health benefits when consumed orally. While these bacteria are generally considered safe, there is a lack of comprehensive data regarding their potential to cause disease. Bacteria belonging to the *Lactobacillus* genus complex rarely cause disease in immunocompetent individuals. Some species have been implicated in infections and have been hypothesized to contain virulence factors that enable them to cause infections more effectively in vulnerable groups. Therefore, it is important to understand factors that could cause disease in susceptible populations that may be present in potential probiotics. This study aims to investigate the genetic similarities between selected lactic acid bacteria (LAB) and pathogenic bacteria. Specifically, the study aims to compare the genes associated with virulence found in pathogenic bacteria, through an existing database, with those found in LAB. Through comparison of these genetic similarities, the study aims to gain insights into the role of ‘virulence and pathogenic factors’ found in disease-causing bacteria that are present in selected LAB. The research can help improve our understanding of the potential risks of probiotics and aid in the development of safer probiotic products for vulnerable populations.

## Introduction

Micro-organisms from the *Lactobacillus* genus complex (LGC), occurring naturally or intentionally introduced, are commonly found in fermented food items and are extensively utilized as probiotic dietary supplements [1]. These bacteria inhabit the oral cavity, digestive system and female reproductive and urinary tract in humans [1]. The majority of lactic acid bacteria are considered suitable for supplement use and oral consumption due to their documented history of safety [2]. The European Food Safety Authority has identified a list of species presumed safe for oral consumption under the 'Qualified Presumption of Safety' concept introduced in 2007 [2]. This list serves as a justifiable basis for food safety, backed by extensive empirical studies and reviews. Similarly, the procedures for assessing food safety in the United States involve the generally recognized as safe (GRAS) regulation [2]. Despite having a list of probiotics considered safe, strain-specific testing must still be employed, as lactobacilli can, in some instances, lead to infections such as endocarditis, bacteremia, neonatal meningitis, dental caries, intraabdominal abscess, pulmonary infections, pyelonephritis and pleuropneumonia [1]. Lactobacilli rarely cause disease in immunocompetent individuals but can cause infections when risk factors and underlying conditions are present (e.g. diabetes mellitus, pre-existing structural heart disease, total parenteral nutrition, organ transplantation, HIV infection and steroid use) [1]. The species frequently implicated in the pathology include *Lacticaseibacillus rhamnosus* and *Lacticaseibacillus paracasei*, both of which harbour potential virulence factors. These factors include the production of enzymes that degrade human glycoproteins and proteins that adhere to extracellular proteins like fibronectin, fibrinogen and collagen. Certain strains can aggregate human platelets, while others can bind fibrinogen. These functions assist Gram-positive pathogens in evading the immune system and fostering platelet aggregation, consequently leading to infections like endocarditis [1].

The potential pathogenesis in patients is multifactorial, including probiotic translocation facilitated by a leaky or damaged intestinal barrier, the existence of virulence factors and harmful metabolites predisposing to opportunistic infections and metabolic disturbances, horizontal gene transfer of antibiotic resistance (AR) genes from probiotics to pathogenic gut bacteria, and an exacerbated immune response elicited through cytokine production [3]. Pathogenic bacteria have the capacity to acquire antibiotic resistance genes from probiotics through horizontal gene transfer. In instances where the probiotic displays intrinsic resistance, the implication is that all antibiotic resistance genes are inherent to all strains within that species [4]. In contrast, when species traditionally susceptible to a drug exhibit resistance, it is considered as acquired resistance [4]. The latter may arise from the acquisition of exogenous DNA or inherent mutations within indigenous genes. Acquired resistance is frequently perceived with a high potential for horizontal spread [4].

Pathogenicity of organisms is characterized by the ability to colonize, invade and damage the host, causing illness [5]. Most pathogens utilize a combination of two properties to induce disease: (1) toxicity, representing the degree to which a substance can cause harm, and (2) invasiveness, the ability to penetrate the host and spread [5]. The ultimate outcome of the disease process depends on the balance between the microbe’s pathogenicity and the host’s immune response status [5]. In healthy individuals, opportunistic pathogens cannot initiate infections as they lack the necessary mechanisms of toxicity and invasiveness to overcome the immune system [5]. In some cases, opportunistic pathogens can cause infections in the setting of a weakened immune response, an altered microbiota or dysbiosis, and invasive procedures/medical devices [5].

The intricate crosstalk between the host and the gut microbiota is essential for maintaining intestinal homeostasis. This balance between different species within the microbial community is termed eubiosis, and any disruption of this balance is referred to as dysbiosis [6]. In a setting of eubiosis, or a healthy and balanced microbial ecology the commensal bacteria prevent opportunistic pathogen infection through microbial competition, antimicrobial production, mucosal barrier integrity maintenance and immunomodulation [7, 8]. The healthy commensal bacteria exert their immunomodulatory functions in a variety of ways. For instance, the gut microbiota plays a crucial role in regulating the expansion of specific lymphocyte subsets, particularly T helper 17 (TH17) cells. TH17 cells are vital for host defence, and the production of pro-inflammatory cytokines, IL-17A, IL-17F and IL-22 [6]. Some specific species of commensal bacteria have been reported to induce the generation of regulatory T (Treg cells), recognized for their regulatory role in the immune system, through TGF-beta activation in epithelial cells. Consequently, disruptions in the healthy microbiota often result in issues with immunomodulation and local defenses on the intestinal epithelium, as observed in autoimmune diseases and the increased susceptibility to opportunistic pathogens [6]. For instance, as a consequence of antibiotic use, commensal micro-organisms are often killed in the process. This results in a reduction of micro-organism-mediated innate immune defenses, allowing residual antibiotic-resistant opportunistic pathogens to inhabit the mucosal surface, such as cases of *C. difficile* infections [8].

The ability of the host to discriminate between pathogenic and commensal bacteria is still poorly understood. It is believed that the sequestration of indigenous microflora by surface epithelium inhibits TLR activation by commensal bacteria [8]. In contrast, pathogenic bacteria possess virulence factors that aid them in traversing the epithelial barrier, allowing recognition by TLRs expressed on dendritic cells and macrophages [8]. Conventional virulence factors include protein toxins and enzymes, cell-surface structures, capsular polysaccharides, lipopolysaccharides and outer membrane proteins, with each playing a direct role in the progression of the disease [9]. The general concept of bacterial pathogenesis is dependent upon host susceptibility and bacterial infectivity [10]. Virulence factors enable bacteria to successfully invade the host, induce disease and circumvent host defenses [10]. These factors can be categorized as adherence factors, invasion factors, capsules, exotoxins, endotoxins and siderophores [10]. Bacterial invasion happens because of complex interactions between a prokaryotic cell and the host target cell. The general steps happen as follows: (1) adherence to eukaryotic cells, (2) entry of the bacteria into the body, (3) avoidance of host immune mechanism, (4) tissue damage and functional impairment and colonization of host tissues and (5) resistance to antibacterial agents [10]. The presence of virulence factors within the genome, however, does not inherently predict their potential to be virulent or cause disease. In some cases, a mutation in a virulence factor from a pathogenic strain may attenuate the pathogenicity. Virulence factors may also be present in attenuated and even avirulent strains [11].

Understanding the mechanisms that enable pathogens to cause diseases is key in treating pathogenic infections and may predict the potential dangers of candidate probiotic strains. Candidate strains are often evaluated for their safety by assessing resistance to antibiotics, and checking for the presence of antibiotic resistance genes, and virulence genes [12]. Additionally, the haemolytic activity, as well as the bile salt hydrolase activity, is often determined at the strain level; however, their importance in probiotic safety is still unclear [2]. In this study, five candidate probiotic strains, *Limosilactobacillus mucosae* LM1 (CP011013.1), *Lactiplantibacillus plantarum* SK 151 (CP030105.1), *Lactiplantibacillus plantarum* BS25 (JAVBIR000000000), *Limosilactobacillus fermentum* SK 152 (CP016803.1) and *Lactobacillus johnsonii* PF01 (CP024781.1) were screened against a database of genes that play a role in infections and host colonization in pathogenic bacteria. These strains were also subjected to antimicrobial resistance (AR) screening and identification. These strains were chosen because of their notable probiotic potential [3, 13–21]. To date, there is no extensive study exploring the potential pathogenicity of the five strains. It is important to screen for potential genetic factors that cause disease in a human host.

## Methodology

### Genome and annotations

The RAST annotation pipeline was used to annotate and view the genomic data of all strains. The genome sequences of LM1, SK151, SK152 and PF01 were downloaded directly from the NCBI GenBank, while that of BS25 was sequenced by the Philippine Genome Center [13]. Genome annotations ran through the Rapid Annotation using Subsystem Technology tool kit (RASTtk) version 1.3.0 pipeline with the following default settings: preserve gene calls disabled, automatically fix errors enabled, fix frameshifts disabled and backfill gaps enabled [22].

### Virulence factor gene mining

A local command line blast search was conducted using blast +software version 2.2.30+. The genome sequences of LM1, SK151, BS25, SK152 and PF01, previously annotated, were utilized, and blast searches were conducted against the Virulence Factor Database (VFDB) containing protein sequences from a core dataset that includes experimentally verified virulence factor genes [23]. The following parameters of blast were applied, with an e-value threshold of 1e-5, max thread of 4, and a maximum target sequence set to 1. Subsequently, the blast outputs were filtered for hits that met a minimum identity of 70 %. In addition, the SEED viewer in the RAST annotation pipeline lists all possible virulence factors according to their database and these were tabulated.

### Antibiotic resistance gene mining

The annotated genes of the strains were queried against the Comprehensive Antibiotic Resistance Database (CARD) using the Resistance Gene Identifier (RGI) web portal version RGI 6.0.3, CARD 3.2.8 using the default parameters: perfect and strict hits only, exclude nudge, and high-quality coverage, to identify antibiotic resistance (AR) genes that exhibit potential for horizontal gene transfer [24]. Additionally, the five strains were also run through ResFinder 4.0 to determine acquired AR genes using default parameters and a 90 % ID and 60 % minimum length threshold [25].

### Genome visualization and comparisons

Visualization of the genomes and annotated genes were visualized and compared using Artemis and the Artemis comparison tool version 18.2.0, and the SEED viewer linked with the RASTtk (version 1.3.0) annotation pipeline [22, 26].

## Results and discussion

### Gene annotation reveals genes associated with virulence, disease and defense

**Fig. 1.**
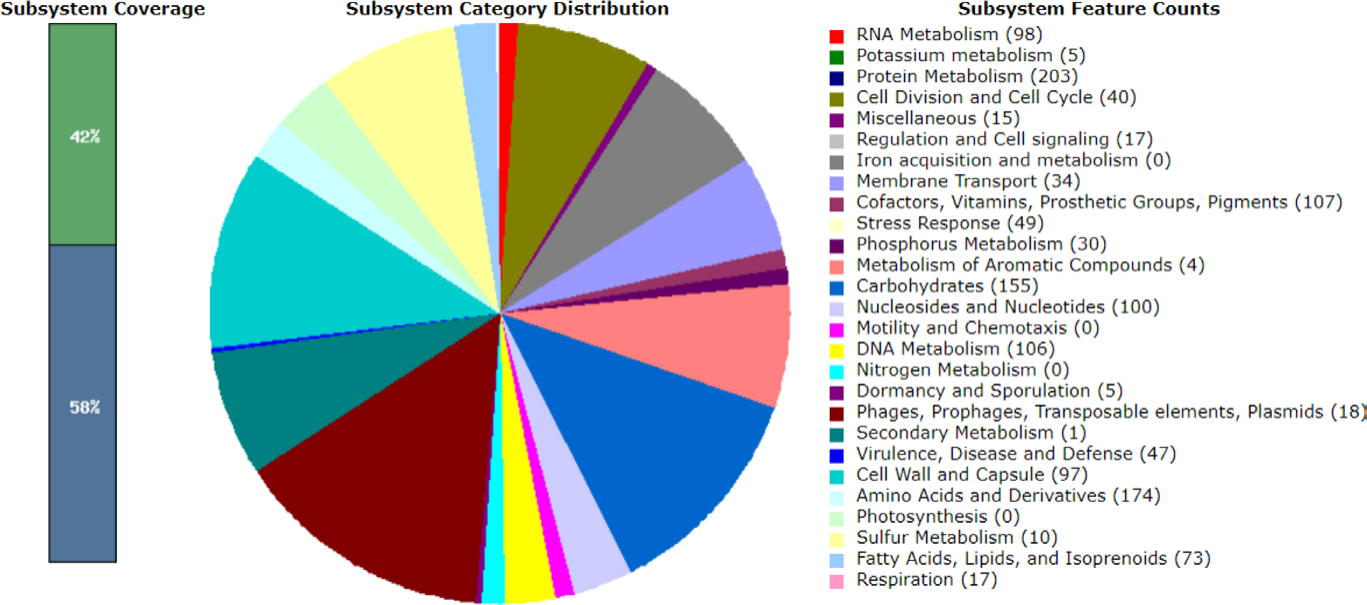
Gene map of *Limosilactobacillus mucosae* LM1.

**Fig. 2.**
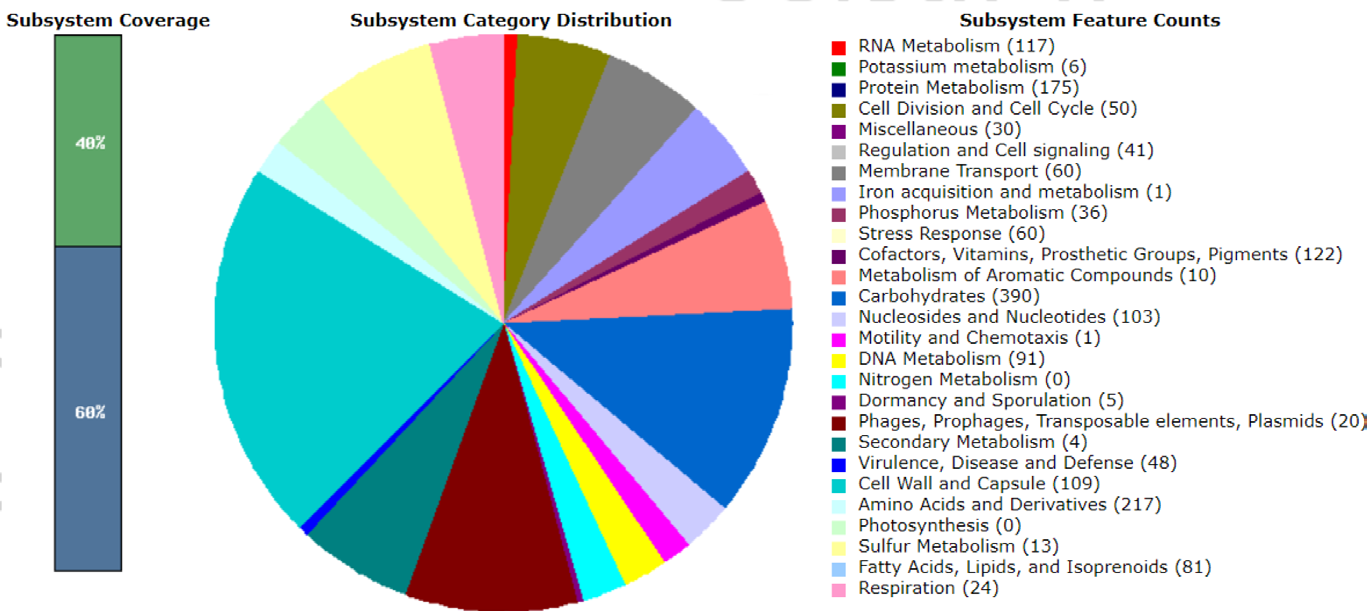
Gene map of *Lactiplantibacillus plantarum* SK151.

**Fig. 3.**
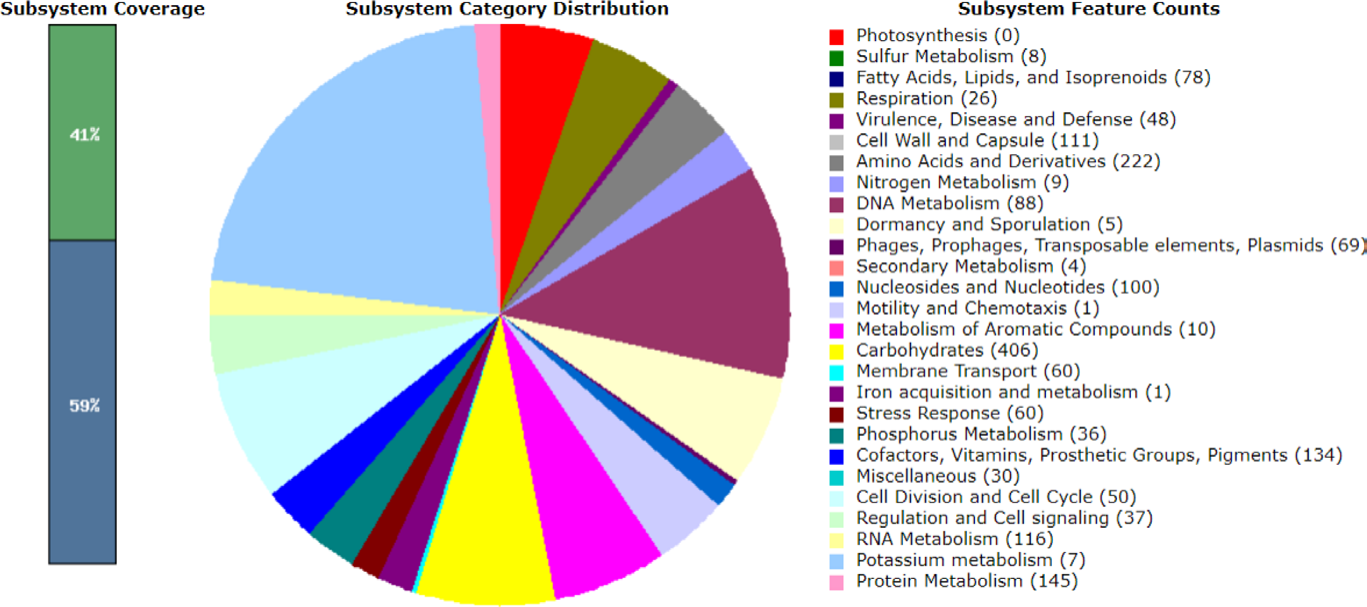
Gene map of *Lactiplantibacillus plantarum* BS25.

**Fig. 4.**
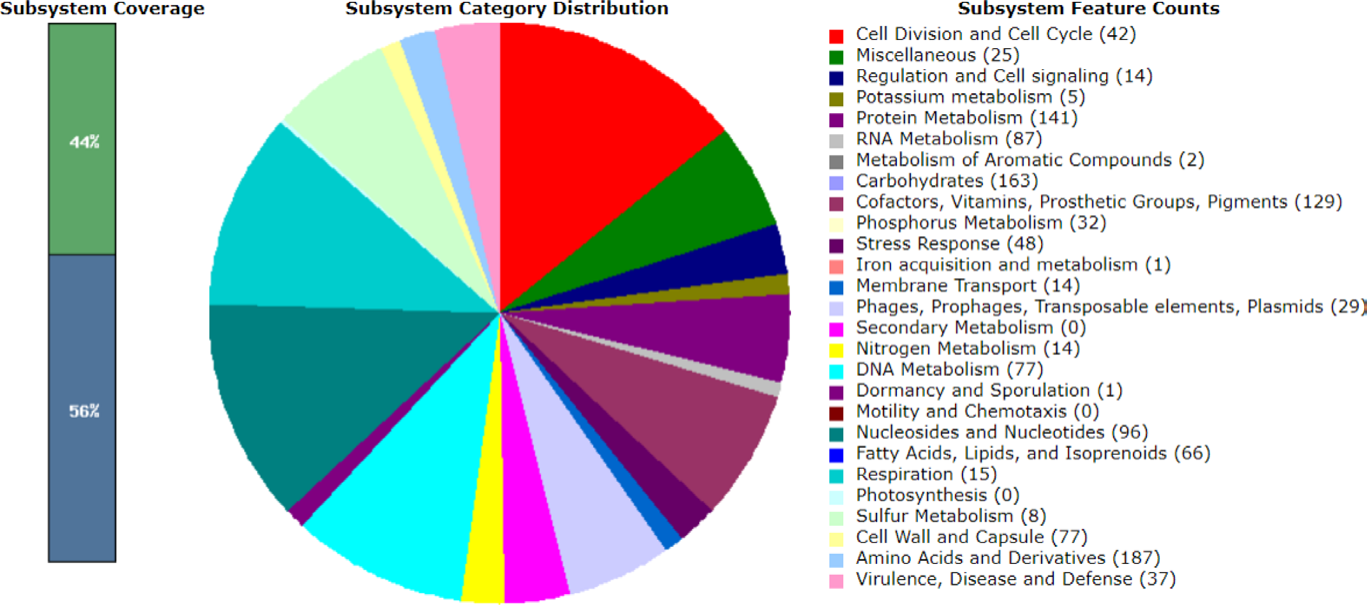
Gene map of *Limosilactobacillus fermentum* SK152.

**Fig. 5.**
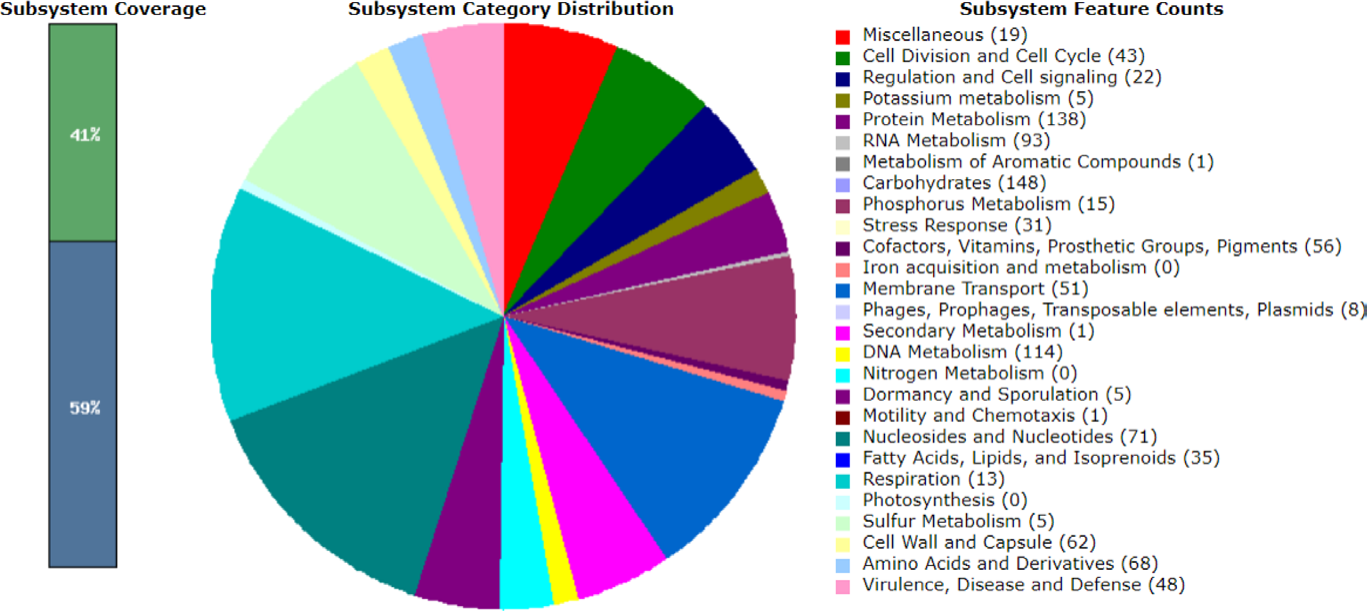
Gene map of *Lactobacillus johnsonii* PF01.

Gene annotation was performed using the RASTtk (version 1.3.0) pipeline, and genes associated with virulence, disease and defence were identified and visualized using the SEED viewer [22]. A total of 47, 48, 48, 37 and 48 associated genes were categorized for LM1, SK151, BS25, SK152 and PF01, respectively. Figs 1–5 show the gene map of the five strains and the grouping of the annotated genes into specific categories. The predicted genes were categorized using the SEED viewer (Table 1). The majority of the genes were categorized under resistance to antibiotics and toxic compounds. Notably, SK151 contains multiple genes related to beta-lactamase resistance, which may raise safety concerns particularly in the possibility of horizontal gene transfer to pathogenic bacteria.

**Table 1.** List of classified virulence genes found in *Limosilactobacillus mucosae* LM1, *Lactiplantibacillus plantarum* SK 151, *Lactiplantibacillus plantarum* BS25, *Limosilactobacillus fermentum* SK 152 and *Lactobacillus johnsonii* PF01 annotated using the RAST annotation pipeline SEED viewer

Virulence, disease and defence gene categories		Strain and no. of genes				
		LM1 (47)	SK151 (48)	BS25 (48)	SK152 (37)	PF01 (48)
Adhesion		2	2	2	2	2
Toxins and superantigens		0	0	0	0	0
Resistance to antibiotics and toxic compounds	Copper homeostasis	11	6	6	5	7
	Bile hydrolysis	1	3	3	0	5
	Cobalt-zinc-cadmium resistance	3	5	4	3	2
	Mercuric reductase	2	0	0	0	2
	Mercury resistance operon	1	0	0	0	1
	Tetracycline resistance, ribosome protection type	3	2	2	2	4
	Resistance to fluoroquinolones	4	4	4	4	4
	Tetracycline resistance, ribosome protection type, too	3	2	2	2	4
	Cobalt resistance	0	0	0	0	0
	Multi-drug-resistance efflux pumps	5	5	4	6	1
	Beta-lactamase	1	7	6	2	5
	Aminoglycoside adenylyltransferases	0	0	0	0	1
	Arsenic resistance	0	0	3	0	0
Invasion and intracellular resistance	*Mycobacterium* virulence operon involved in protein synthesis (SSU ribosomal proteins)	4	4	4	4	4
	*Mycobacterium* virulence operon involved in DNA transcription	2	2	2	2	3
	*Mycobacterium* virulence operon involved in protein synthesis (LSU ribosomal proteins)	3	3	3	3	3
	*Mycobacterium* virulence operon involved in an unknown function with a Jag Protein and YidC and YidD	2	3	3	2	0

Bile salt hydrolase (BSH) encoding genes were identified in LM1, SK151, BS25 and PF01 strains with one, three, three and five homologues, respectively. The presence of BSH is crucial for the survival of probiotics, as host bile acids can act as bacteriostatic agents, negatively impacting intestinal flora by dissolving bacterial membranes [27]. This enzyme can detoxify bile, facilitating the colonization of lactic acid bacteria in the gastrointestinal tract [28]. Many probiotic strains harbour multiple BSH homologues, providing a potential survival advantage. Each homologue may exhibit distinct responses to various bile types or exposure durations, ultimately maximizing survival [28]. Currently, there is no literature implicating any potential for pathogenicity associated with the presence of BSH encoding genes, but its importance may be hypothesized to play a role in documented cases of cholecystitis associated with lactic acid bacteria [29]. In addition, no virulence factors involved in human host damage, haemolysis and toxin production were detected. The possible factors that could play a role in pathogenicity (e.g. adhesion mechanism and bile salt hydrolase activity) contribute to the survival of micro-organisms in the mammalian gut and such are characteristics of much of the local microbiota. It is therefore not recommended to use these factors as a basis for pathogenicity as they are not *a priori* meaningful measures of virulence [30].

### Presence of survival and virulence factors and their implications on the safety of probiotic candidates

LM1, SK151, BS25, S152 and PF01 were queried against a database downloaded from the Virulence Factor Database (VFDB; http://www.mgc.ac.cn/VFs) core dataset, and command line blast was performed using the annotated sequences [23]. The VFDB core dataset is composed of virulence factors experimentally verified across the literature [23]. A total of 4, 8, 6, 1, and 2 hit/s (excluding repeat hits) were obtained from LM1, SK151, BS25, SK152 and PF01, respectively. The listed hits from the database in Table 2 were tabulated based on percent ID and bit score, omitting repeating genes. All five strains identified the *eno* gene. The *L. plantarum* strains SK151 and BS25 share matches with the following genes: *eno*, *clfA*, *sdrE*, *sdrF* and *sdrG*. The genes identified are characterized by functions related to surface adhesion, immunomodulation and platelet aggregation, and each will be discussed in further detail. No genes were detected related to haemolysis using the RAST annotation as well as the local blast with the VFDB core dataset.

**Table 2.** Top sequence matches of the queried strains against VFDB with their function and potential role in virulence

Gene	Query strain/s	Reference organism	%ID	Function	Role in virulence	Reference/s
(*tufA*) elongation factor Tu	LP BS25 PF01	*Francisella tularensis* subsp. holarctica FTNF002-00	72.926	A G protein that catalyses the binding of aminoacyl-tRNA to the A-site of the ribosome inside living cells.	Ef-Tu binds immune system regulators (e.g. Factor H, substance P, plasminogen) increasing virulence and immune system evasion.	[32]
		*Mycoplasma mycoides* subsp. mycoides SC str. PG1	75.217			
(*eno*) phosphopyruvate hydratase	BS25	*Streptococcus suis* 98HAH33	75.313	Catalyses the conversion of 2-phosphoglycerate into phosphoenolpyruvate.	Binds with the human plasmin zymogen plasminogen (Plg) and subsequently enhances the formation of proteolytic plasmin activity.	[37]
	PF01	*Streptococcus agalactiae* A909	73.811			
	SK152	*Streptococcus suis* 98HAH33	79.897			
	LM1	*Streptococcus pneumoniae* Hungary19A-6	75.868			
	SK151	*Streptococcus suis* 98HAH33	75.235			
(*clfA*) Clumping factor A	LPBS25	*Staphylococcus aureus* subsp. aureus MRSA252	77.148	It is the ligand for the integrin alphaIIb/beta3 on the surface of platelets. This binding of fibrinogen to the integrin receptor on activated platelets results in platelet aggregation and the formation of platelet-fibrin thrombi. ClfA exhibits fibrinogen-binding characteristics like those of the platelet integrin alphaIIb/beta3.	Adherence.ClfA and ClfB bind to different sites in fibrinogen. ClfA binds to the gamma-chain. ClfA through its fibrinogen-binding function is a mediator *of S. aureus-*induced platelet aggregation	[47]
	SK151	*Staphylococcus aureus subsp*. aureus N315	77.277			
(*gspB*) platelet binding protein	LM1	*Streptococcus gordonii* str. M99	71.279	Binds human platelets through its interaction with a platelet membrane receptor, glycoprotein (GP) Ibα	Plays a role in the pathogenesis of infective endocarditis through the mediation of platelet binding.	[41, 54]
(*hsa*) accessory Sec-dependent serine-rich glycoprotein adhesin	LM1	n/a	70.299	Sialic acid binding haemagglutinin and mediates the agglutination of platelets	Mediates adhesion to platelets and implicated in the pathogenesis of infective endocarditis. Aggregation of streptococci in the presence of saliva in the context of tooth colonization.	[42, 55]
(*rfbB*) dTDP-glucose 4,6-dehydratase	LM1	*Streptococcus gordonii* str. Challis	74.458	Involved in the biosynthesis of dTDP-rhamnose and immunoregulation	Upregulated in the setting of an attenuated the immune response of macrophages	[35]
(*sdrC*) Ser-Asp rich fibrinogen-binding bone sialoprotein-binding protein	SK151	*Staphylococcus aureus* subsp.	77.307	Attachment to host tissues and synthetic surfaces coated with plasma proteins	Molecular determinant in staphylococcal biofilm formation triggers bacterial adhesion to abiotic surfaces	[45]
(*sdrD*) Ser-Asp rich fibrinogen-binding bone sialoprotein-binding protein	SK151	*Staphylococcus aureus* subsp. aureus Mu50	79.599	Attachment to host tissues and synthetic surfaces coated with plasma proteins	Innate immunity escape, dampening of neutrophil activity	[43]
(*sdrE*) Ser-Asp rich fibrinogen-binding bone sialoprotein-binding protein	SK151	*Staphylococcus aureus* subsp.	79.381	Attachment to host tissues and synthetic surfaces coated with plasma proteins	Complement evasion via the interaction with complement factor H	[46]
	LPBS25	*Staphylococcus aureus* subsp. aureus MW2	79.167			
(*sdrF*) MSCRAMM family adhesin	LPBS25	*Staphylococcus epidermidis* ATCC 12228	78.577	Attachment to host tissues and synthetic surfaces coated with plasma proteins. SdrF in *S. epidermidis* binds type I collagen and Dacron.	Plays a role in the initial colonization stage of *S. epidermidis* infections associated with complications arising from prosthetic device implantation.	[56]
	SK151	*Staphylococcus epidermidis* ATCC 12228	79.085			
(*sdrG*) MSCRAMM family adhesin	LPBS25	*Staphylococcus epidermidis* ATCC 12228	82.552	Attachment to host tissues and synthetic surfaces coated with plasma proteins. Binds to bone sialoprotein and fibrinogen.	Attachment to fibrinogen on a plastic surface, which can initiate device-related infections.	[47]
	SK151	*Staphylococcus epidermidis* ATCC 12228	82.439			
(*sdrH*) MSCRAMM-like protein SdrH	SK151	*Staphylococcus epidermidis* ATCC 12228	82.143	Attachment to host tissues and synthetic surfaces coated with plasma proteins. Involved in the initial attachment of bacterium on the surface during biofilm formation.	Involved in biofilm formation	[42]

Table 2 Top sequence matches of the queried strains against the VFDB with their function and potential role in virulence.

It has been mentioned previously that the presence of haemolytic genes must be assessed in candidate probiotic strains to evaluate their safety. The presence of haemolytic activity in probiotics is undesirable as it can cause anaemia and oedema in the host. It is important to screen for haemolysin produced by a strain as it breaks down host cells, releasing iron-containing compounds like haemoglobin [31]. If the strain exhibits β-hemolytic activity, it is considered harmful, whereas γ-haemolytic and α-haemolytic activities are generally considered safe [31]. The blast results against the VFDB for the five candidate strains showed no genes related to haemolysis but *in vitro* testing is still recommended.

Immunomodulation and surface adhesion are some of the key functions of lactic acid bacteria to establish initial gut colonization. The results from the blast reveal an identical gene, *tufa*, present in lactic acid bacteria strains BS25 and PF01, analogous to that of pathogenic bacteria *Tularensis* subsp. *holarctica* and *Mycoplasma mycoides* subsp. *mycoides,* respectively. The *tufa* gene encodes an elongation factor Tu (Ef-Tu) G protein, which facilitates the binding of aminoacyl-tRNA to the A-site of the ribosome within living cells [32]. In pathogenic Gram-positive and Gram-negative bacteria, Ef-Tu showcases a range of diverse roles. These functions include binding to immune system regulators, enhancing virulence, promoting immune evasion, facilitating invasion of host cells, and interacting with Mreb to regulate cell shape. In addition, Ef-Tu can be fragmented via proteolytic processing, acting as molecular decoys that, in turn, promote immune evasion [32]. In contrast, in some *Lactobacillus* species, Ef-Tu performs various functions, such as attachment to human intestinal cells, host immunomodulation, mucin binding and actin bindin [33]. A study by Granato *et al*. Granato et al. (2004) on *Lactobacillus johnsonii* reported that Ef-Tu, in this species, binds to human intestinal cells and mucins in a pH-dependent manner, suggesting its contribution to gut colonization [34]. Additionally, this function enables lactic acid bacteria to exert beneficial effects by competitively adhering to the intestinal mucosa, thereby excluding potentially pathogenic bacteria and preventing infections [34]. The protein RfbB from the reference strain *Streptococcus gordonii* str. *Challis*, was matched with a protein found in the LM1 strain. RfbB, also known as dTDP-glucose 4,6 dehydratase, is involved in the biosynthesis of dTDP-rhamnose and immunoregulation. It has been shown to be upregulated in the setting of an attenuated immune response of macrophages [35]. In *Salmonella typhimurium*, it has been reported that the function of RfbB is to catalyse the synthesis of both O-antigen and the enterobacterial common antigen [36]. These lipopolysaccharides play a crucial role in maintaining the structural integrity of the outer membranes of Gram-negative bacteria. The functional loss of RfbB, using a gene deleted mutant of *S. typhimurium*, resulted in defects in outer cell-wall permeability, leading to hypersensitivity to bile and cell-wall-targeting antibiotics. Additionally, the loss of the protein led to a reduced production of pro-inflammatory cytokines [36]. This highlights the potential role of RfbB in cell-wall integrity, immunomodulation and virulence in *S. typhimurium* [36], and it can be hypothesized to function similarly in the probiotic strain LM1.

The strains BS25, PF01, SK152 and LM1 have hits with various *Streptococcus* species on the *eno* gene, which codes for the protein phosphopyruvate hydratase belonging to the enolase family. Enolase is a metalloenzyme that catalyses the reversible conversion of 2-phospho-d-glycerate (2-PGE) to phosphoenolpyruvate, a pivotal reaction in glycolytic pathways across diverse organisms, including bacteria. Surface-associated enolase has been observed to interact with the human host in streptococcal infection cases. In the pathogenesis of *S. pneumoniae* and *S. pyogenes*, it engages with human plasminogen (Plg), enhancing proteolytic plasmin activity, which plays a pivotal role in the progression of pneumococcal and group A streptococcal infections [37]. In contrast, a gene found in the study by Castaldo *et al*. Castaldo et al. (2009) revealed an *enoA1* gene, coding for EnoA1 alpha-enolase, which plays a role in fibronectin binding [38]. Fibronectin is present in the extracellular matrix of intestinal epithelial cells, and surface adhesion through fibronectin binding is essential for probiotic transit and potential integration with the intestinal mucosa [38]. Currently, no literature can be found regarding the role of enolase in BS25, PF01, SK152 and LM1 strains concerning Plg interactions. It has been established that efficient probiotic *Lactobacillus* strains share certain characteristics with pathogens, including survival and adherence factors crucial for competition with pathogens. Existing data also suggests that probiotic lactobacilli interact with immune cell receptors and influence the functions of epithelial cells. Some of these interactions parallel those observed in pathogens but are not present in commensal and resident bacteria. While some pathogenic bacteria use certain mechanisms to evade the immune system or downregulate the immune response, lactobacilli utilize similar mechanisms but instead produce immune response-promoting effects [39].

The aggregation of blood platelets by bacteria is believed to contribute to the pathogenesis of infective endocarditis, which results from the deposition of platelet fibrin clots on the endothelial surface of the heart [40]. Platelet aggregation has been more extensively studied and reported in *Staphylococcus aureus*, *Streptococcus sanguis* and group B streptococci but studies on Lactobacilli are limited. A study by Harty *et al*. Harty et al. (1993) reported that certain lactobacilli can aggregate human platelets with particularly increased frequency in *L. rhamnosus* strains [40]. The gene *GspB*, producing a cell-wall-anchored glycoprotein, was identified in strain LM1. This gene encodes a cell-surface protein crucial for platelet binding in *Streptococcus gordonii* strain M99 [41]. GspB shares identity with a protein called Hsa, expressed by *S. gordononii* Challis [41]. GspB facilitates the binding of various carbohydrates containing sialic acid in either α(2-3) or α(2-6) linkages [41]. A notable difference between GspB and Hsa lies in their localization: despite its significant role in adherence to sialic acid, most of the Hsa expressed in *S. gordononii* Challis remains within the cytoplasm [41]. Hsa is also recognized as a sialic-binding haemagglutinin and has been demonstrated to have roles in platelet agglutination [41, 42]. The potential of LM1 to induce blood platelet aggregation and cause infective endocarditis is still uncertain and requires further testing.

The strains SK151 and BS25 both belong to the *Lactiplantibacillus planatrum* strain, and have been shown to have genes related to Microbial Surface Components Recognizing Adhesive Matrix Molecules (MSCRAMMs) identical to those found in *Staphylococcus* species. *S. aureus* has been known to have these MSCRAMMs, which allow it to survive in a variety of tissue cells [43]. MSCRAMMs are adhesins characterized by the presence of a minimum of two IgG-like folds and utilize a ligand-binding mechanism known as the 'close, dock, lock and latch (CDLL)’ mechanism. The MSCRAMMs recognize and bind to extracellular matrix proteins, initiating the pathogenic process. Typically, MSCRAMMs are known to interact with fibrinogen, fibronectin, neurexin and IgGs. Staphylococcal MSCRAMMs, belonging to the Clf-Sdr protein family, include clumping factor A (ClfA), clumping factor B (ClfB) and the Sdr proteins, such as SdrC, SdrD and SdrE in *S. aureus*, and SdrF and SdrG in *S. epidermidis*. In *S. aureus*, both ClfA and ClfB serve as fibrinogen-binding proteins and are upregulated during biofilm growth. ClfA induces bacterial clumping when combined with fibrinogen [44]. Additionally, SdrC facilitates bacterial intercellular interactions and contributes to biofilm formation. Even in the non-pathogenic bacterium *Lactococcus lactis*, there is evidence suggesting that SdrC promotes bacterial adhesion to abiotic surfaces. Additionally, studies suggest that the involvement of SdrC in biofilm formation varies based on the strain background [45]. SdrD plays crucial roles in both colonization and infection by promoting bacterial survival in blood and inhibiting human neutrophil activity. In addition, it inhibits innate immune-mediated bacterial killing independently of other *S. aureus* proteins. In the case of non-pathogenic bacteria, introducing recombinant SdrD and expressing it heterologously in *Lactococcus lactis* have both been found to enhance its survival in human blood [43]. The protein SdrD has been shown to exhibit an immune evasion tactic in *S. aureus*. When it binds to human complement factor H (CFH) via the CDLL mechanism, it captures the C-terminal tail of CFH, and the process sequesters CFH on the bacterial surface, resulting in complement evasion [46]. Some strains of *S. epidermidis* can attach to fibrinogen immobilized in a plastic surface, which increases the likelihood of device-related infections when the device is implanted in the human body, and it has been shown that the protein SdrG plays a significant role in this process through adherence to fibrinogen. Regarding its role in lactic acid bacteria, studies have shown that a non-pathogenic strain *Lactococcus lactis* MG1363 made to express high levels of SdrG through an expression vector showed that these strains strongly adhered to immobilized fibrinogen [47]. Lastly, the SdrH protein is implicated in the initial attachment of the bacterium during biofilm formation on a surface. It was observed to be upregulated during the initial 4 h of biofilm formation in *S. epidermidis*, although the exact mechanisms for this process are yet to be elucidated [48]. Based on the presence of Sdr encoding genes in *L. plantarum* species it could be hypothesized that they are capable of biofilm formation as well as an enhanced tendency to survive in the bloodstream. It is also important to note that both *L. plantarum* BS25 and SK151 share multiple Sdr proteins found in both *S. aureus* and *S. epidermidis*, indicating their propensity for possible biofilm formation and device-related adhesion. However, there is not enough evidence to suggest the likelihood of bloodstream infections associated with *L. plantarum* especially in immunocompetent individuals. It was noted above that engineered *Lactococcus lactis* with elevated expressions of *sdrC* and *sdrD* had improved surface adhesion to abiotic surfaces and improved survival in blood [43, 45], respectively, but these alterations do not necessarily entail any increased risk of bacteremia. One explanation for the low pathogenicity and virulence of lactobacilli in healthy human hosts can be attributed to their lack of tissue destruction and 'true' virulence factors. Consequently, their interaction with the host is generally favourable [39]. The majority of the genes presented above have roles in biofilm formation, surface aggregation, immune detection and immunomodulation and have not yet been fully explored in all five strains. To date, no literature thoroughly explores the role and regulation of these genes in pathogenicity for lactic acid bacteria.

Although multiple hits were derived from the VFDB as presented above, it does not predict the pathogenicity of the five LAB strains. The common functions of the genes recognized through the VFDB are related to survival, surface adhesion and recognition of the host immune response. As mentioned previously, the mechanisms of pathogenicity of *Lactobacillus* spp. are often through the breakdown of human glycoproteins, proteins that bind extracellular proteins, interactions with fibrinogen, and the ability to aggregate platelets. Genes identified using the VFDB are not implicated in the observed mechanisms of lactic acid bacteria pathology and are mostly related to survival and surface adhesion. Land *et al*. Land et al. (2005) reported two patients who were administered probiotic bacilli later experienced bacteremia and sepsis linked to *Lactobacillus* species [49]. Subsequent molecular fingerprinting indicated that the *Lactobacillus* strain identified in the blood sample was identical to the probiotic strain consumed. This case then concludes that even evaluated and approved *Lactobacillus* strains have the propensity to cause disease in certain populations. It is important to mention that the cases mentioned previously are paediatric cases that have concomitant diseases (i.e. heart valve disease and UTI). Therefore, it may be a question of host susceptibility rather than the presence of acquired survival factors of *Lactobacillus* that cause disease. This does not however discourage the use of probiotics but rather caution its use on certain populations [49]. Assessing the potential virulence factors in bacterial groups without recognized and documented pathogenic members remains a challenge [49].

In this study, all virulence factors were compared against known virulence factors found in recognized pathogenic bacteria (e.g. *S. aureus*). No definitive pathogenicity-related genes have been identified in *Lactobacillus* or *Bifidobacterium* species used as probiotics [50]. Cases of sepsis in vulnerable populations linked to lactobacilli lack conclusive evidence from clinical isolates, suggesting no species-specific properties that can lead to infection [50]. In addition, the current obstacle in predicting the pathogenicity of the identified survival and defence factors lies in the fact that these genes have not been thoroughly studied in the context of probiotic infections. Identifying variants of these genes and how they are regulated in members of the LGC that cause infections and bacteremia may yield valuable insights into the process of screening for the safety of potential probiotics. The possibility of enhancing, silencing and selectively regulating these genes for safety remains to be explored.

### Antibiotic resistance (AR) genes and horizontal gene transfer (HGT)

One of the potential dangers of introducing a foreign probiotic is the possible integration of antibiotic resistance through horizontal gene transfer toward more pathogenic bacteria. Allard *et al*. Allard et al. (2002) conducted a study to assess the antibiotic susceptibility of more than 182 *Lactobacillus* type strains and compared these findings with genome-wide annotations of antibiotic resistance genes (AR genes) [51]. They discovered that most had genes encoding resistance to aminoglycosides, tetracycline, erythromycin, clindamycin and chloramphenicol [51]. According to the European Food Safety Authority (EFSA), the possibility of transfer of resistance genes to pathogenic bacteria is related to the genetic basis of resistance. The likelihood for intrinsic resistance to undergo horizontal spread is believed to be low [4].

The five strains were also analysed using the Resistance Gene Identifier tool found in Comprehensive Antibiotic Resistance Database (CARD). In addition, the genomes were also analysed using an online bioinformatics tool, ResFinder 4.0, for predictions of phenotypes from AR genes [25].

Further observation of the genomes of the five strains revealed resistance genes to tetracycline (*tetW*) found on both strains LM1 and PF01. The strain PF01 also had a match to an AR gene related to erythromycin (*ermT*) on strain PF01. These findings are not uncommon for probiotics. A study by Campedelli *et al*. Campedelli et al. (2019) showed that many lactobacilli are antibiotic-resistant [52]. Most *Lactobacillus* strains are intrinsically resistant to aminoglycosides, ciprofloxacin and trimethoprim [52]. Additionally, other reports suggest that some lactic acid bacteria have intrinsic resistance to bacitracin, kanamycin, teicoplanin, vancomycin and beta-lactams [30]. Analysis of strains LM1, BS25, SK151 and SK152 revealed glycopeptide resistance gene clusters: *vanT* gene in *vanG* cluster, *vanY* gene in *vanB* cluster, *vanY* gene in *vanB* cluster and *vanT* gene in *vanG* cluster, respectively. Strains from fermented foods have been shown to contain acquired resistances to erythromycin, tetracycline, chloramphenicol and clindamycin [52]. Studies have also revealed that more than 75 % of antibiotic resistance genes present in the human flora of screened individuals are specific to tetracycline, macrolides and beta-lactams [30]. Results from ResFinder 4.0 confirmed the presence of tetracycline and erythromycin resistance genes in PF01. In addition, *ermT* and *tetW* hits were also identified as acquired AMR gene hits in PF01. Similarly, *tetW* was identified as an acquired AMR gene in LM1. No acquired AMR gene hits were identified for BS25, SK151 and SK152. In the context of possible horizontal gene transfer to pathogenic bacteria, it is important to consider that most horizontal transfer events in the bacterial chromosome are often deleterious and there is a low mechanistic probability of HGT occurrence and may take months to years to occur [53].

## Conclusion and future perspectives

The study gathered information on genes found in selected probiotic strains that may cause harm in humans using RAST gene annotation, VFDB, AR gene mining (ResFinder), and manual identification through the related literature. The majority of the genes categorized under ‘virulence, disease and defence’ identified using the RAST pipeline are associated with bacterial resistance to drugs and environmental conditions. This study is still limited by the fact that the virulence factors in the database are based on known pathogenic bacteria and may exhibit different mechanisms of pathogenicity. To date, there is no known ‘true’ pathogenic LAB, which serves as an obstacle in predicting virulence factors specific to LAB and its respective species. There is no absolute pathogenic strain representative of the five species/strains that can help narrow down significant virulence factors for these potential probiotics. The genes mined using the VFDB mostly reported genes associated with biofilm formation, surface adherence, platelet aggregation, survival and host immune modulation and would be misleading to be considered as virulence factors in the context of probiotics. No genes were detected associated with toxins and superantigens. All five strains contain potential intrinsic antibiotic resistance genes related to tetracycline and erythromycin. The potential pathogenicity of the five LAB strains is possibly dependent on the host immune response. It is important to note that even probiotic strains approved and commercialized have been known to cause bacteremia in susceptible individuals, but no universal genetic markers have been identified that can predict their pathogenicity. Mutational analysis of the mined survival factors with a special attention to fibrinogen-binding genes should be performed, as they are commonly associated with a disease process in isolated cases. It would also be beneficial to have a database of cases with lactic acid bacteremia to create a comprehensive database of gene variants that can cause pathology.

## References

[1] Rossi F, Amadoro C, Colavita G (2019). Members of the *Lactobacillus* genus complex (LGC) as opportunistic pathogens: a review. Microorganisms.

[2] Binda S, Hill C, Johansen E, Obis D, Pot B (2020). Criteria to qualify microorganisms as “probiotic” in foods and dietary supplements. Front Microbiol.

[3] Kothari D, Patel S, Kim S-K (2019). Probiotic supplements might not be universally-effective and safe: a review. Biomed Pharmacother.

[4] Gabriele A, Georges B, Andrew C (2018). Guidance on the assessment of bacterial susceptibility to antimicrobials of human and veterinary importance. EFS2.

[5] Beceiro A, Tomás M, Bou G (2013). Antimicrobial resistance and virulence: a successful or deleterious association in the bacterial world?. Clin Microbiol Rev.

[6] Al-Rashidi HE (2022). Gut microbiota and immunity relevance in eubiosis and dysbiosis. Saudi J Biol Sci.

[7] Belkaid Y, Hand TW (2014). Role of the Microbiota in Immunity and Inflammation. Cell.

[8] Buffie CG, Pamer EG (2013). Microbiota-mediated colonization resistance against intestinal pathogens. Nat Rev Immunol.

[9] Huang H, Liu N, Guo H, Liao S, Li X (2012). L-Carnitine Is an Endogenous HDAC Inhibitor Selectively Inhibiting Cancer Cell Growth In Vivo and In Vitro. PLoS ONE.

[10] Peterson Johnny W, Baron S (1996). Med Microbiol.

[11] Yongqun H (2014). Bacterial Whole-Genome Determination and Applications. vol. 1–3.

[12] Casarotti SN, Carneiro BM, Todorov SD, Nero LA, Rahal P (2017). In vitro assessment of safety and probiotic potential characteristics of *Lactobacillus* strains isolated from water buffalo mozzarella cheese. Ann Microbiol.

[13] Charina B, Francisco E, Leslie D (2004). Identification and characterization of bacteriocinogenic *Lactobacillus* plantarum Bs25 isolated from Balao-Balao, a locally fermented rice-shrimp mixture from the Philippines. Philipp Agric Sci.

[14] Amoranto Mia Beatriz C, Ju KO, Bagon Bernadette B (2018). Complete genome sequence of *Lactobacillus plantarum* Sk151 isolated from Kimchi. Korean J Microbiol.

[15] Balolong MP, Chae JP, Kang D-K (2016). Expression and characterisation of neopullulanase from *Lactobacillus mucosae*. Biotechnol Lett.

[16] Kim SH, Song JH, Kim J, Kang D-K (2020). Characterisation of a lysophospholipase from *Lactobacillus mucosae*. Biotechnol Lett.

[17] Hoon KS (2020). Park Hye Kyun, Hwang in-Chan, Kang Dae-Kyung. antimicrobial substance of *Lactobacillus johnsonii* Pf01. J Dairy Sci Biotechnol.

[18] Yoon LJ, Pajarillo Edward Alain B, Jeong KM, Pyo CJ, Kyung KD (2013). Proteomic and transcriptional analysis of *Lactobacillus johnsonii* Pf01 during bile salt exposure by Itraq shotgun Proteomics and quantitative RT-PCR. J Proteome Res.

[19] Pajarillo EAB, Kim SH, Lee J-Y, Valeriano VDV, Kang D-K (2015). Quantitative proteogenomics and the reconstruction of the metabolic pathway in *Lactobacillus mucosae* LM1. Korean J Food Sci Anim Resour.

[20] Valeriano VD, Parungao-Balolong MM, Kang D-K (2014). In vitro evaluation of the mucin-adhesion ability and probiotic potential of *Lactobacillus mucosae* LM1. J Appl Microbiol.

[21] Valeriano VD, Bagon BB, Balolong MP, Kang D-K (2016). Carbohydrate-binding specificities of potential probiotic *Lactobacillus* strains in porcine jejunal (IPEC-J2) cells and porcine mucin. J Microbiol.

[22] Brettin T, Davis JJ, Disz T, Edwards RA, Gerdes S (2015). RASTtk: a modular and extensible implementation of the RAST algorithm for building custom annotation pipelines and annotating batches of genomes. Sci Rep.

[23] Liu B, Zheng D, Jin Q, Chen L, Yang J (2019). VFDB 2019: A comparative pathogenomic platform with an interactive web interface. Nucleic Acids Res.

[24] Alcock BP, Raphenya AR, Lau TTY, Tsang KK, Bouchard M (2020). CARD 2020: antibiotic resistome surveillance with the comprehensive antibiotic resistance database. Nucleic Acids Res.

[25] Bortolaia V, Kaas RS, Ruppe E, Roberts MC, Schwarz S (2020). ResFinder 4.0 for predictions of phenotypes from genotypes. J Antimicrob Chemother.

[26] Carver T, Harris SR, Berriman M, Parkhill J, McQuillan JA (2012). Artemis: an integrated platform for visualization and analysis of high-throughput sequence-based experimental data. Bioinformatics.

[27] Hofmann AF (1999). The continuing importance of bile acids in liver and intestinal disease. Arch Intern Med.

[28] Begley M, Hill C, Gahan CGM (2006). Bile salt hydrolase activity in probiotics. Appl Environ Microbiol.

[29] Tena D, Martínez NM, Losa C, Fernández C, Medina MJ (2013). Acute acalculous cholecystitis complicated with peritonitis caused by *Lactobacillus plantarum*. Diagn Microbiol Infect Dis.

[30] Imperial I, Ibana JA (2016). Addressing the antibiotic resistance problem with probiotics: reducing the risk of its double-edged sword effect. Front Microbiol.

[31] Amoah K, Dong X, Tan B, Zhang S, Kuebutornye FKA (2021). In vitro assessment of the safety and potential probiotic characteristics of three *Bacillus* strains isolated from the Intestine of hybrid grouper (Epinephelus fuscoguttatus♀ × Epinephelus lanceolatus♂). Front Vet Sci.

[32] Harvey KL, Jarocki VM, Charles IG, Djordjevic SP (2019). The diverse functional roles of elongation factor Tu (EF-Tu) in microbial pathogenesis. Front Microbiol.

[33] Iebba V, Totino V, Gagliardi A, Santangelo F, Cacciotti F (2016). Eubiosis and dysbiosis: the two sides of the microbiota. New Microbiol.

[34] Granato D, Bergonzelli GE, Pridmore RD, Marvin L, Rouvet M (2004). Cell surface-associated elongation factor Tu mediates the attachment of *Lactobacillus johnsonii* NCC533 (La1) to human intestinal cells and mucins. Infect Immun.

[35] Liu T, Yang R, Zhou J, Lu X, Yuan Z (2021). Interactions between *Streptococcus gordonii* and *Fusobacterium nucleatum* altered bacterial transcriptional profiling and attenuated the immune responses of macrophages. Front Cell Infect Microbiol.

[36] Subhashish C, Pip B, Joel J (2023). Functional loss of rffG and rfbB, Encoding dTDP-glucose 4,6-Dehydratase, changes colony morphology, cell shape, motility and virulence in Salmonella Typhimurium. BioRxiv.

[37] Antikainen J, Kuparinen V, Lähteenmäki K, Korhonen TK (2007). Enolases from Gram-positive bacterial pathogens and commensal lactobacilli share functional similarity in virulence-associated traits. FEMS Immunol Med Microbiol.

[38] Castaldo C, Vastano V, Siciliano RA, Candela M, Vici M (2009). Surface displaced alfa-enolase of *Lactobacillus plantarum* is a fibronectin binding protein. Microb Cell Fact.

[39] Lebeer S, Vanderleyden J, De Keersmaecker SCJ (2010). Host interactions of probiotic bacterial surface molecules: comparison with commensals and pathogens. Nat Rev Microbiol.

[40] Harty DW, Patrikakis M, Hume EB, Oakey HJ, Knox KW (1993). The aggregation of human platelets by Lactobacillus species. J Gen Microbiol.

[41] Bensing BA, López JA, Sullam PM (2004). The *Streptococcus gordonii* surface proteins GspB and Hsa mediate binding to sialylated carbohydrate epitopes on the platelet membrane glycoprotein Ibalpha. Infect Immun.

[42] Takahashi Y, Konishi K, Cisar JO, Yoshikawa M (2002). Identification and characterization of hsa, the gene encoding the sialic acid-binding adhesin of *Streptococcus gordonii* DL1. Infect Immun.

[43] Wang X, Ge J, Liu B, Hu Y, Yang M (2013). Structures of SdrD from *Staphylococcus aureus* reveal the molecular mechanism of how the cell surface receptors recognize their ligands. Protein Cell.

[44] O’Brien L, Kerrigan SW, Kaw G, Hogan M, Penadés J (2002). Multiple mechanisms for the activation of human platelet aggregation by *Staphylococcus aureus*: roles for the clumping factors ClfA and ClfB, the serine-aspartate repeat protein SdrE and protein A. Mol Microbiol.

[45] Barbu EM, Mackenzie C, Foster TJ, Höök M (2014). SdrC induces *Staphylococcal* biofilm formation through a homophilic interaction. Mol Microbiol.

[46] Zhang Y, Wu M, Hang T, Wang C, Yang Y (2017). *Staphylococcus aureus* SdrE captures complement factor H’s C-terminus via a novel “close, dock, lock and latch” mechanism for complement evasion. Biochem J.

[47] Hartford O, O’Brien L, Schofield K, Wells J, Foster TJ (2001). The Fbe (SdrG) protein of *Staphylococcus epidermidis* HB promotes bacterial adherence to fibrinogen. Microbiology.

[48] Shivaee A, Mohammadzadeh R, Shahbazi S, Pardakhtchi E, Ohadi E (2019). Time-variable expression levels of mazF, atlE, sdrH, and bap genes during biofilm formation in Staphylococcus epidermidis. AMicr.

[49] Land MH, Rouster-Stevens K, Woods CR, Cannon ML, Cnota J (2005). *Lactobacillus* sepsis associated with probiotic therapy. Pediatrics.

[50] Sanders ME, Akkermans LMA, Haller D, Hammerman C, Heimbach J (2010). Safety assessment of probiotics for human use. Gut Microbes.

[51] Allard STM, Beis K, Giraud MF, Hegeman AD, Gross JW (2002). Toward a structural understanding of the dehydratase mechanism. Structure.

[52] Campedelli I, Mathur H, Salvetti E, Clarke S, Rea MC (2019). Genus-wide assessment of antibiotic resistance in *Lactobacillus* spp. Appl Environ Microbiol.

[53] Townsend JP, Bøhn T, Nielsen KM (2012). Assessing the probability of detection of horizontal gene transfer events in bacterial populations. Front Microbiol.

[54] Xiong YQ, Bensing BA, Bayer AS, Chambers HF, Sullam PM (2008). Role of the serine-rich surface glycoprotein GspB of *Streptococcus gordonii* in the pathogenesis of infective endocarditis. Microb Pathog.

[55] Bensing BA, Gibson BW, Sullam PM (2004). The Streptococcus gordonii platelet binding protein GspB undergoes glycosylation independently of export. J Bacteriol.

[56] Toba FA, Visai L, Trivedi S, Lowy FD (2013). The role of ionic interactions in the adherence of the Staphylococcus epidermidis adhesin SdrF to prosthetic material. FEMS Microbiol Lett.

